# The role of national trainees associations in pandemic times

**DOI:** 10.1192/j.eurpsy.2021.1740

**Published:** 2021-08-13

**Authors:** M.J. Santos, A. Samouco, Z. Azvee, A. Seker, T. Mogren, M. Pinto Da Costa

**Affiliations:** 1 Mental Health Department, Hospital Prof. Doutor Fernando Fonseca, Amadora, Portugal; 2 Department Of Psychiatry And Mental Health, Unidade Local de Saúde do Norte Alentejano, Portalegre, Portugal; 3 Mental Health Service, Galway Roscommon Mental Health Service, Galway, Ireland; 4 Child And Adolescent Psychiatry, Erciyes University Hospital, Kayseri, Turkey; 5 Psychiatry Center Södertälje, Health Care Services Stockholm County, Stockholm, Sweden; 6 Unit For Social And Community Psychiatry, WHO Collaborating Centre for Mental Health Services Development, Queen Mary University of London, London, United Kingdom

**Keywords:** COVID-19, leadership, trainees

## Abstract

**Introduction:**

The challenges posed by the COVID-19 pandemic were many and daunting. Almost overnight, the lives of millions of people all over Europe was disrupted and people had to adapt to a completely new situation. Healthcare personal were amongst the ones most affected by it, whether by changes in their everyday work routine or by being the people directly in charge of responding to the demands of the pandemic. Trainees are an indispensable part of healthcare personal and, as a result, they were vastly affected by the pandemic.

**Objectives:**

Discussing the role of National Trainees Associations (NTAs) in pandemic times, including how they dealt with the new challenges, their successes and hardships, and their steps going forward.

**Methods:**

Gathering of institutional information about the work of the Portuguese, Irish and Turkish NTAs during the COVID-19 pandemic. Critical appraisal of each of their contributions and projects.

**Results:**

There were impacts to trainees’ clinical work, formative activities and even personal lives. Amidst this turmoil, NTAs were precious institutions. Their objective is to represent the trainees of each country, looking for formative opportunities and linking trainees. During the pandemic, the work developed by the NTAs increase in importance, serving as a beacon of important information and as intermediaries in a number of discussions. They also aimed to minimize the impact on formative activities, whether by providing recommendations or by organizing some form of educational activity.

**Conclusions:**

We provide some national examples – Portugal, Ireland and Turkey – and draw comparisons and lessons from each one.

**Disclosure:**

No significant relationships.

